# Vancomycin-Loaded Silk Fibroin/Calcium Phosphate/Methylcellulose-Based In Situ Thermosensitive Hydrogel: A Potential Function for Bone Regeneration

**DOI:** 10.3390/gels10110695

**Published:** 2024-10-25

**Authors:** Premchirakorn Phewchan, Artit Laoruengthana, Pratthana Chomchalao, Supaporn Lamlertthon, Waree Tiyaboonchai

**Affiliations:** 1 Department of Pharmaceutical Technology, Faculty of Pharmaceutical Sciences and Center of Excellence for Innovation in Chemistry, Naresuan University, Phitsanulok 65000, Thailand; premchirakorn@gmail.com; 2 Center of Excellence for Innovation in Chemistry (PERCH-CIC), Department of Chemistry, Faculty of Science, Mahidol University, Bangkok 10400, Thailand; 3 Department of Orthopedics, Faculty of Medicine, Naresuan University, Phitsanulok 65000, Thailand; artitlao@gmail.com; 4 College of Medicine and Public Health, Ubon Ratchathani University, Ubon Ratchathani 34190, Thailand; pratthana.c@ubu.ac.th; 5 Department of Microbiology and Parasitology, Faculty of Medical Sciences, Naresuan University, Phitsanulok 65000, Thailand; supapornl@nu.ac.th; 6 The Center of Excellence in Medical Biotechnology, Naresuan University, Phitsanulok 65000, Thailand

**Keywords:** silk fibroin, calcium phosphate, MC3T3-E1 cells, osteogenic differentiation, osteomyelitis, bone regeneration

## Abstract

This study explores the efficacy of a vancomycin-loaded silk fibroin/calcium phosphate/methylcellulose-based in situ thermosensitive hydrogel (VM-SF/CaP/MC) in promoting the osteogenic differentiation of preosteoblast cells. Three VM-SF/CaP/MC formulations with varying low (L) and high (H) concentrations of silk fibroin (SF) and calcium phosphate (CaP) were prepared: VM-HSF/LCaP/MC, VM-LSF/HCaP/MC, and VM-HSF/HCaP/MC. These hydrogels significantly enhanced MC3T3-E1 cell migration and proliferation in a dose- and time-dependent manner, achieving complete cell migration within 48 h. In addition, they significantly promoted alkaline phosphatase activity, collagen content, and mineralization in MC3T3-E1 cells, indicating their potential for osteogenesis. Among the hydrogel formulations, the VM-HSF/HCaP/MC hydrogel, with high SF and CaP content, demonstrated superior potential in promoting the osteogenic differentiation of MC3T3-E1 cells. It exhibited the highest ALP activity (11.13 ± 0.91 U/mg protein) over 14 days, along with increased collagen content (54.00 ± 1.71 µg/mg protein) and mineralization (15.79 ± 1.48 mM) over 35 days. Therefore, this formulation showed a promising candidate for clinical application in localized bone regeneration, particularly in treating osteomyelitis.

## 1. Introduction

Osteomyelitis (OM) poses a challenge in orthopedics due to associated bone necrosis, frequent recurrence, prolonged hospitalizations, and considerable negative impact on patient health as well as financial consequences. A significant proportion of OM cases are attributed to *Staphylococcus* species, including antibiotic-resistant strains such as methicillin-resistant *Staphylococcus aureus* (MRSA) [1,2]. The management of OM involves the radical debridement of necrotic and infected bone tissue, followed by extensive intravenous antibiotic (ATB) treatment for 4–6 weeks [3]. However, long-term systemic ATB treatment could result in systemic toxicity, such as nephrotoxicity or ototoxicity [4,5].

Administering localized antimicrobial therapy, traditionally with ATB-loaded polymethylmethacrylate (PMMA), helps increase bactericidal effect while reducing the risk of extensive long-term ATB [6,7]. However, the PMMA is unable to establish osseous connections with the host bone or facilitate osteogenesis and angiogenesis processes [8,9], and subsequent surgery is usually required to remove the PMMA after ATB elution. Currently, an optimal OM therapeutic approach has focused on biomaterials that provide dual function, either antibacterial effect or bone regeneration stimulation. Antibiotic-loaded in situ hydrogels (ATB-ISHs) have emerged as a promising and superior therapeutic strategy due to their unique properties, including injectable delivery, thermoresponsive formation, sustained antibiotic release, biodegradability, and bone regeneration promotion. As an offering of ATB-ISHs properties, we successfully developed a novel locally injectable vancomycin-loaded silk fibroin/calcium phosphate/methylcellulose-based in situ thermosensitive hydrogel (VM-SF/CaP/MC) [10]. This formulation was achieved by optimizing the combination of silk fibroin (SF), methylcellulose (MC), and calcium phosphate (CaP) content, which influence the physicochemical properties of hydrogels. SF is an attractive biomaterial candidate for in situ hydrogels due to its biocompatibility, hydrolytic biodegradability, and effective facilitation of new bone formation [11]. However, pure SF solutions exhibit a slow gelation time at body temperature (~1–2 days) [12,13]. Thus, blending SF with thermosensitive polymers like MC can significantly accelerate the gelation time of SF hydrogels by promoting the β-sheet conformation of SF [12,14]. Incorporating CaP, a bio-osteostimulative agent, can enhance the mechanical properties of hydrogels and, more importantly, promote bone regeneration [15,16]. Additionally, owing to the antibacterial properties of ATB-ISHs, vancomycin (VM), which is a glycopeptide ATB effective against Gram-positive bacteria as well as methicillin-resistant *Staphylococcus aureus* (MRSA) was selected as a model drug [17].

Consequently, our research findings demonstrate that the developed VM-SF/CaP/MC hydrogel maintains a liquid state at room temperature, facilitating precise injection into target sites. Upon exposure to physiological temperature (37 °C), the hydrogel undergoes rapid gelation and forms a well-defined microstructure within 7 min. Importantly, VM-SF/CaP/MC exhibits significant biodegradability (>60%) within 35 days under 37 °C, eliminating the necessity for additional surgical procedures. Moreover, VM-SF/CaP/MC functions as a dual-purpose local drug delivery system. Firstly, the hydrogel ensures sustained VM release over 35 days, maintaining therapeutic levels at the infection site and effectively combating *Staphylococcus aureus*. Secondly, the incorporated CaP component transforms into hydroxyapatite (HAP) at a physiological pH of 7.4, suggesting its potential to promote bone regeneration. However, further investigation is warranted to conclusively establish the bone regeneration potential of VM-SF/CaP/MC, particularly regarding their osteostimulation function.

In the present study, we conducted a comprehensive biological evaluation of VM-SF/CaP/MC employing the MC3T3-E1 preosteoblast cell line. This assessment included biocompatibility, cell migration, cell proliferation, and osteogenic activity, with the primary aim of elucidating the potential of VM-SF/CaP/MC to promote in vitro osteogenic differentiation for bone regeneration.

## 2. Results

### 2.1. Cell Viability

**Figure 1:**
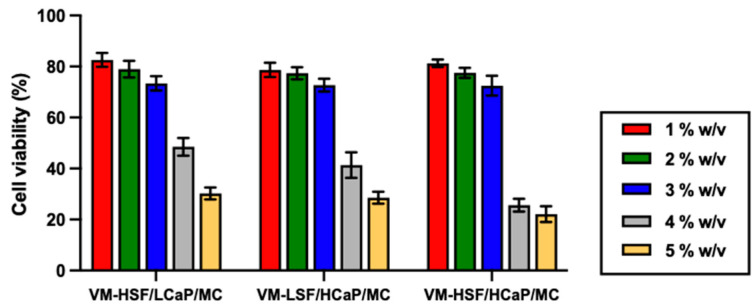
Cell viability of MC3T3-E1 cells after treatment with 1–5% *w*/*v* of extracted VM-SF/CaP/MC for 24 h. The values are expressed as mean ± SD, (*n* = 3).

To evaluate the osteogenic differentiation potential of VM-SF/CaP/MC, the optimal concentration of extracted VM-SF/CaP/MC on cell viability was conducted using the CCK-8 assay based on dehydrogenase activity in the viable cells. According to ISO 10993-5:2009 [18], a cell viability of >70% was classified as nontoxic. The effect of the extracted VM-SF/CaP/MC at various concentrations (1–5% *w*/*v*) on MC3T3-E1 cells is shown in Figure 1. The cell viability exhibited a dose-dependent behavior. After 24 h incubation, there was no cytotoxicity observed at all formulations of VM-SF/CaP/MC concentrations of ≤3% *w*/*v*, whereas at concentrations of 4 and 5% *w*/*v*, a decline in cell viability was observed, suggesting cytotoxicity. Thus, based on the cell viability assays, the extracted VM-SF/CaP/MC at 1–3% *w*/*v* was used in the cell migration, proliferation, and in vitro osteogenic differentiation studies.

### 2.2. Cell Migration

**Figure 2:**
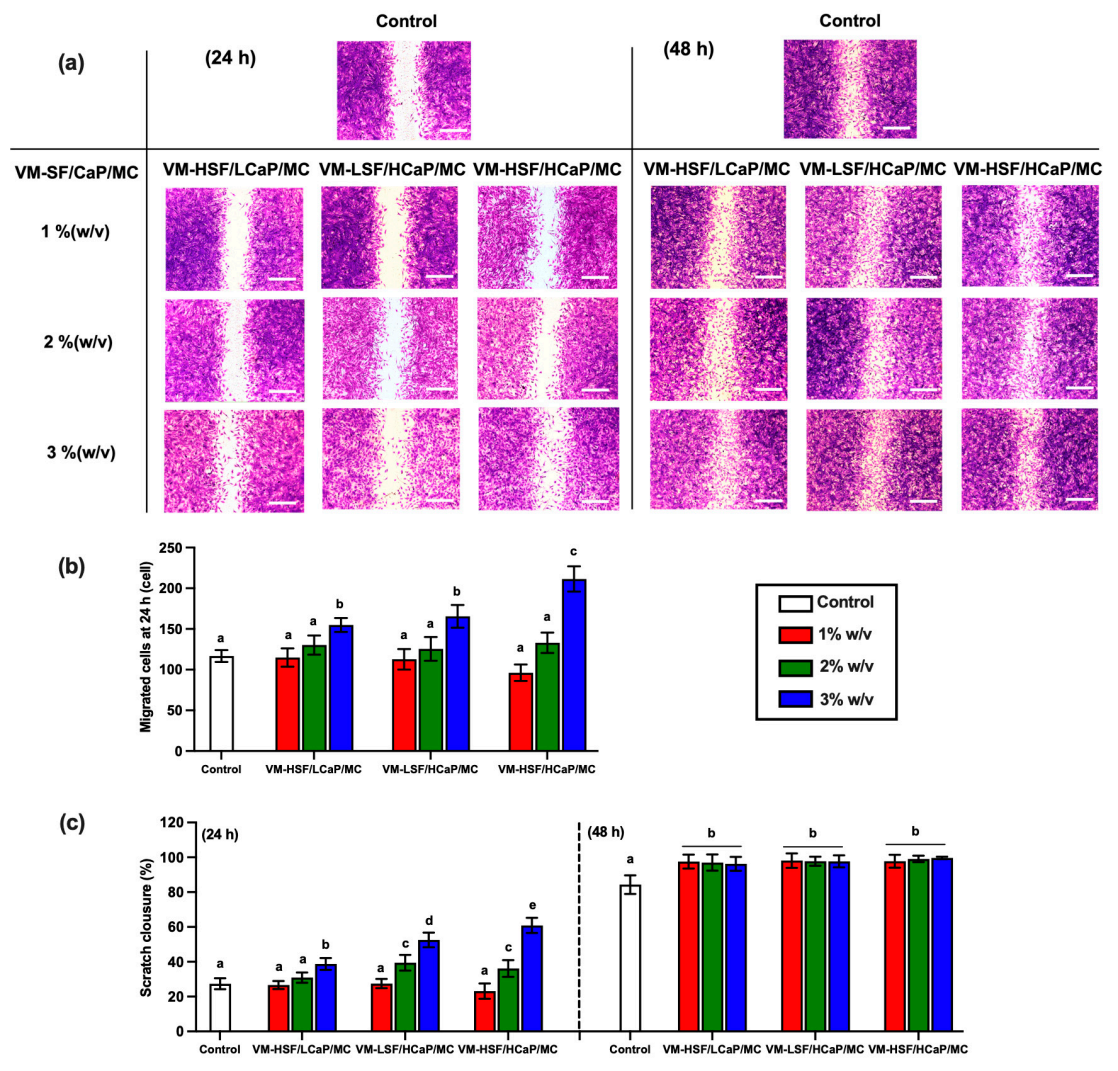
The migration ability of MC3T3-E1 exposed to different concentrations of VM-SF/CaP/MC for 24 and 48 h; (**a**) the migratory capacity of MC3T3-E1 cells at 24 h and 48 h; (**b**) the number of migrated cells on the scratch area at 24 h; and (**c**) the percentage of scratch closure after 24 and 48 h. Data represent means ± SD (*n* = 3). Different letters indicate statistically significant differences between groups of each time point (*p* < 0.05). One representative image from three independent experiments is shown at 5× magnification with a scale bar of 200 μm.

The scratch wound healing assay was performed to evaluate the potential of VM-SF/CaP/MC for inducing cell migration. The effect of the extracted VM-SF/CaP/MC at various concentrations (1–3% *w*/*v*) on MC3T3-E1 cell migration is shown in Figure 2. After 24 h of incubation, VM-SF/CaP/MC enhanced the migration of MC3T3-E1 cells, compared to the control group (Figure 2a). The cell-free area decreased as the extracted VM-SF/CaP/MC concentration increased. Notably, all extracted VM-SF/CaP/MC at 3% *w*/*v* showed significantly greater cell migration to the wound area and displayed the least cell-free areas. The number of migrated cells to the wound area was observed in the following order: VM-HSF/HCaP/MC > VM-HSF/LCaP/MC ≈ VM-LSF/HCaP/MC, (Figure 2b). In addition, the % scratch closure at 24 h of VM-HSF/HCaP/MC, VM-HSF/LCaP/MC, and VM-LSF/HCaP/MC was 60.89 ± 4.33%, 38.73 ± 3.42%, and 52.52 ± 4.20%, respectively, (Figure 2c). These results indicate that the high content of CaP and SF in the VM-SF/CaP/MC formulations promote the migration of MC3T3-E1 cells over the wound area during the initial step of wound healing. Interestingly, all concentrations of the extracted VM-SF/CaP/MC exhibited nearly 100% wound closure after 48 h incubation, while the control was 84.33 ± 5.34%. This result suggests that CaP and SF in the hydrogel formulations could facilitate cell migration for bone repair.

### 2.3. Cell Proliferation

**Figure 3:**
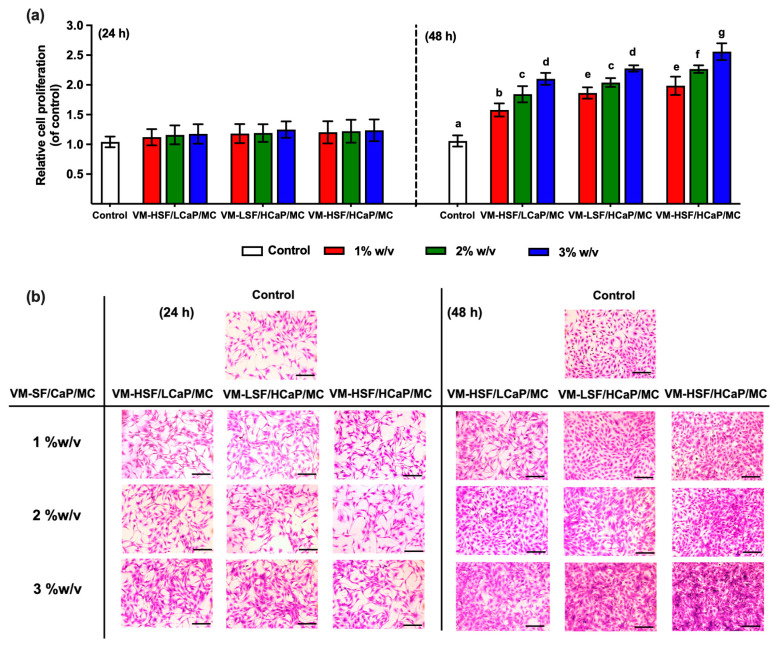
Proliferation of MC3T3-E1 preosteoblast cells after treatment with various concentrations of VM-SF/CaP/MC for 24 and 48 h: (**a**) relative of cell proliferation (RCP); and (**b**) crystal violet staining images of MC3T3-E1 cells. Data represent means ± SD (*n* = 3). Different letters indicate statistically significant differences between groups (*p* < 0.05). One representative image from three independent experiments is shown at 10× magnification with a scale bar of 200 μm.

The effect of VM-SF/CaP/MC on the proliferation of MC3T3-E1 preosteoblast cells, was assessed using the CCK-8 assay, and crystal violet staining compared to the control group (media without the hydrogel). Figure 3a illustrates that the relative cell proliferation (RCP) of MC3T3-E1 cells exposed to VM-SF/CaP/MC responded in a time- and dose-dependent manner. At 24 h of cultivation, there was no significant difference in RCP between the VM-SF/CaP/MC and the control. Interestingly, after 48 h of culturing, RCP increased when the concentration of VM-SF/CaP/MC increased, where the 3% *w*/*v* VM-HSF/HCaP/MC formulation demonstrated the highest RCP. In addition, crystal violet staining confirmed increased cell proliferation, particularly at the 3% *w*/*v* concentration of VM-SF/CaP/MC, with higher cell densities than the control in a time- and dose-dependent manner. The morphology of MC3T3-E1 cells treated with VM-SF/CaP/MC exhibited a spindle-shaped as a fibroblast-like morphology at both 24 and 48 h of culturing similar to the control group, indicating well-organized cytoskeletons for attachment (Figure 3b). Furthermore, at 48 h of cultivation, the cell populations increased, and cells grew in a multilayer morphology. These results demonstrate that VM-SF/CaP/MC has excellent characteristics to support cell growth and proliferation. Therefore, further studies were conducted on 3% *w*/*v* of each VM-SF/CaP/MC in preosteoblast MC3T3-E1 osteogenic differentiation.

### 2.4. Alkaline Phosphatase (ALP) Activity

**Figure 4:**
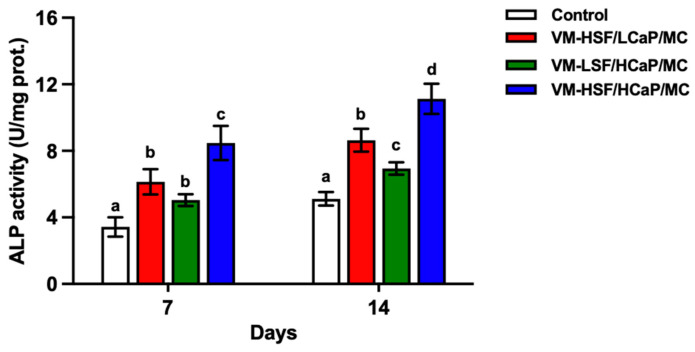
Alkaline phosphatase (ALP) activity of MC3T3-E1 cells after treatment with VM-SF/CaP/MC for 7 and 14 days. The error bars represent mean ± S.D., (*n* = 3); the different letters indicate statistically significant differences between groups at each time point (*p* < 0.05).

ALP, a well-established enzyme produced by osteoblasts, plays an important role in the early stage of osteogenic differentiation. As expected, the cells treated with all VM-SF/CaP/MC formulations significantly produced ALP activity higher than the control in a time-dependent manner, (Figure 4). In particular, on day 14 of culture, VM-HSF/HCaP/MC exhibited the highest ALP activity (11.13 ± 0.91 U/mg protein) followed by VM-HSF/LCaP/MC (8.64 ± 0.69 U/mg protein) and VM-LSF/HCaP/MC (6.94 ± 0.37 U/mg protein), respectively. This result suggests that SF content in the hydrogel formulation had an impact on enhancing ALP activity. It is worth noting that the increase in ALP activity correlated with the increased SF content in the hydrogel formulation.

### 2.5. Collagen Deposition

**Figure 5:**
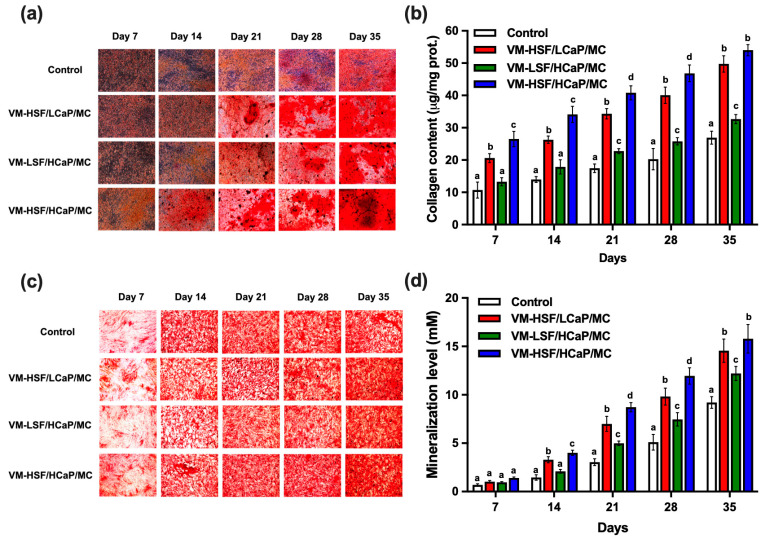
The effect of VM-SF/CaP/MC on collagen deposition and mineralization. MC3T3- E1 cells were incubated with VM-SF/CaP/MC for 35 days. (**a**) Sirius Red (SR) staining of qualitative of collagen deposition; (**b**) the amount of collagen content determined using a colorimetric method; (**c**) Alizarin Red S (ARS) staining of qualitative of the extracellular calcium deposition; and (**d**) the degree of mineralization for 35 days. All data represent the mean ± S.D., (*n* = 3); the different letters indicate statistically significant differences between groups at each time point (*p* < 0.05).

Collagen secretion by osteoblasts is the main organic component of bone tissue. Thus, Sirius Red staining was used to detect the collagen deposition of MC3T3-E1 cells exposed to VM-SF/CaP/MC compared with the control cells at predetermined time points (7, 14, 21, 28, and 35 days). Microscopic observation revealed strong staining on the cells treated with VM-SF/CaP/MC, indicating collagen deposition (Figure 5a). From the quantitative analysis, cells treated with all VM-SF/CaP/MC formulations showed significantly higher collagen content than the control (Figure 5b). All samples exhibited an increase in collagen content as the time increased. At day 35, VM-HSF/HCaP/MC displayed the highest collagen content (54.00 ± 1.71 µg/mg protein) followed by VM-HSF/LCaP/MC (49.76 ± 2.52 µg/mg protein) and VM-LSF/HCaP/MC (36.66 ± 1.39 µg/mg protein), respectively. This result suggests that SF plays a crucial role in promoting collagen synthesis.

### 2.6. Extracellular Matrix Mineralization

Alizarin Red S (ARS) staining was used to assess mineralization. As shown in Figure 5c, no significant calcium deposition was observed on day 7 in all groups. Nevertheless, cells treated with VM-HSF/HCaP/MC presented a large calcium nodule area and red color staining on days 21, 28, and 35. Notably, the control group exhibited fewer calcium nodules at all the testing periods. The validity of this finding was affirmed through the quantitative analysis of the mineralization levels (Figure 5d). The formulations with high SF content (VM-HSF/LCaP/MC and VM-HSF/HCaP/MC) displayed significantly greater mineralization levels than the formulation with low SF content, VM-LSF/HCaP/MC, and the control starting from day 14. The level of mineralization was increased in a time-dependent manner. By day 35, calcium deposition markedly increased for VM-HSF/LCaP/MC and VM-HSF/HCaP/MC, reaching mineralization levels of 14.56 ± 1.20 and 15.79 ± 1.48 mM, respectively. Although VM-LSF/HCaP/MC showed less calcium deposition of 12.21 ± 0.72 mM, it was significantly higher than the control group (9.21 ± 0.60 mM). These findings suggest that VM-SF/CaP/MC could promote calcification in MC3T3-E1 cells for bone regeneration.

## 3. Discussion

**Scheme 1:**
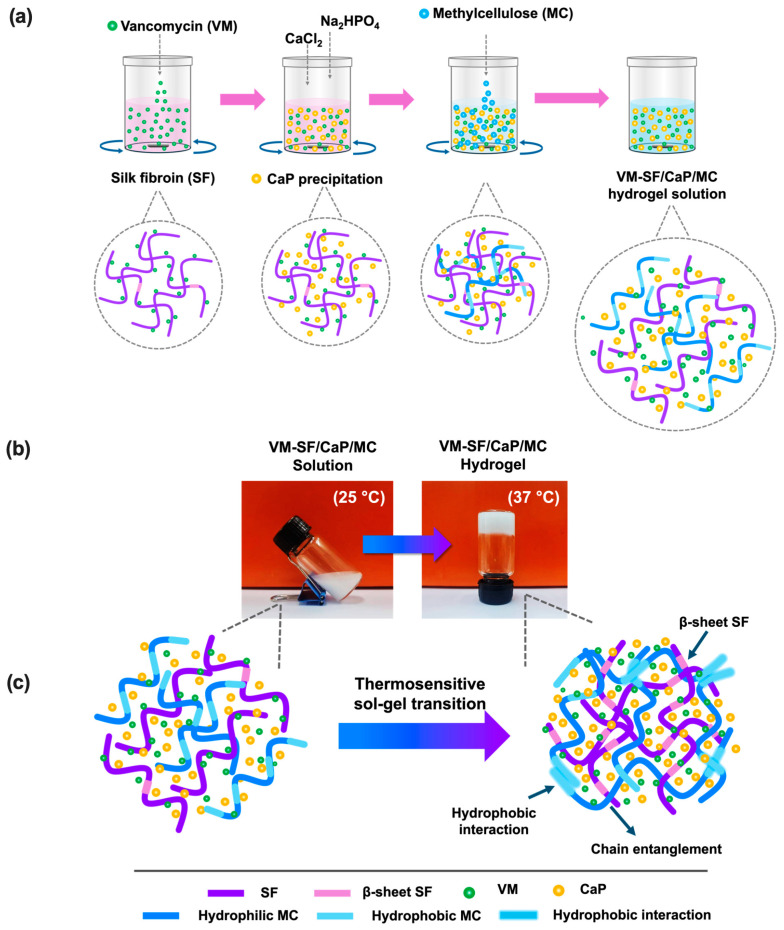
Representation of (**a**) the preparation of VM-SF/CaP/MC hydrogel; (**b**) the optical appearance and thermosensitive sol–gel transition of the VM-SF/CaP/MC solution at 25 °C and its transformation into a hydrogel at 37 °C; and (**c**) the hydrogelation process of the VM-SF/CaP/MC hydrogel as the temperature changes.

The presence of bacteria in osteomyelitis lesions results in the release of osteolytic cytokines and osteonecrosis factors, leading to significant local bone loss and destruction associated with devascularization [19]. Thus, current treatments of osteomyelitis focus on two primary goals: inhibiting bacterial growth and stimulating bone regeneration. Recently, we successfully developed the in situ thermosensitive hydrogel formulation of VM-SF/CaP/MC with different SF and CaP content (VM-HSF/LCaP/MC, VM-LSF/HCaP/MC, and VM-HSF/HCaP/MC) to serve as a versatile platform for localized VM delivery for osteomyelitis treatment [10]. The VM-SF/CaP/MC hydrogel was prepared using the CaP precipitation technique, in which CaP is precipitated into the VM-loaded SF, followed by mixing with MC. The prepared VM-SF/CaP/MC hydrogel displays a white appearance, with CaP evenly dispersed within the solution. At room temperature, the hydrogels exhibit a low viscosity solution, making them easy to administer and applicable for a small and complex infection site. Then, these hydrogels undergo a thermosensitive sol–gel transition, transforming from a liquid solution at room temperature (25 °C) to a hydrogel at body temperature (37 °C) within 5 min, as shown in Scheme 1b.

At 37 °C, the hydrogelation of VM-SF/CaP/MC occurs through the entanglement of SF and MC chains, accompanied by an increase in the β-sheet conformation of SF. Additionally, the salting-out effect of residual ions in the hydrogel solution enhances hydrophobic interactions between MC molecules. VM is entrapped within the hydrogel structure, interacting with the negatively charged SF and the hydrophilic nature of both SF and MC, as illustrated in Scheme 1c. Importantly, the VM-SF/CaP/MC hydrogels exhibited a prolonged VM release for 35 days. The hydrogel release medium was evaluated for its antibacterial efficacy by maintaining therapeutic levels above the minimum inhibitory concentration (MIC) of 2 µg/mL against *S. aureus* ATCC25923 for 35 days [10], highlighting the bactericidal action of VM through the disruption of bacterial cell wall synthesis [20].

Moreover, the prepared VM-SF/CaP/MC hydrogel maintained a pH of ~4.8 to ensure the stability of VM within the hydrogel. Additionally, the CaP was present as dicalcium phosphate dihydrate (DCPD) polymorphism, which could be converted into hydroxyapatite (HAP), a bioactive substance that supports bone formation when pH changes to 7.4 (physiological pH) [10]. Thus, this study investigated the efficacy of these hydrogels in enhancing bone regeneration to fulfill their dual function as antibiotic-loaded hydrogels for treating bacterial infections and promoting bone tissue regeneration.

In this study, preosteoblasts are crucial precursor cells that differentiate into osteoblasts and are responsible for bone formation. In this study, MC3T3-E1 cells were chosen due to their well-defined characteristics and established capacity for osteogenic differentiation, making them a popular selection for in vitro bone research [21,22].

The osteogenic differentiation of the preosteoblast cells involves the following three main stages: (1) preosteoblast cell proliferation, in which cells rapidly divide to increase their numbers and facilitate osteoblast differentiation; (2) matrix maturation, during which cells begin to express specific proteins and deposit an extracellular matrix; and (3) mineralization, where the deposited matrix undergoes calcification, forming mature bone tissue. Each of these stages is crucial for effective bone repair and regeneration [11,23].

The in vitro biological study on VM-SF/CaP/MC aimed to optimize its concentration for osteogenic differentiation study. It was found that all formulations at concentrations of ≤3% *w*/*v* showed no cytotoxicity. However, at concentrations of 4% and 5% *w*/*v*, a decrease in cell viability was observed, suggesting potential cytotoxic effects. This effect may be attributed to the high diffusion of CaP from the VM-SF/CaP/MC, leading to increased cell apoptosis, consistent with previous studies [24,25,26]. Therefore, the VM-SF/CaP/MC hydrogel formulations at concentrations of ≤3% *w*/*v* were approved for testing their effects on cell migration and proliferation.

At the initial stage of bone regeneration, cell migration is a fundamental process in which osteogenic cells, including osteoblasts and osteoprogenitor cells, need to migrate to the site of injury or bone defect affected by the infection to initiate the bone healing process [27]. Hence, the role of VM-SF/CaP/MC in facilitating the migration process of MC3T3-E1 cells was investigated. Subsequently, cell proliferation is a crucial process to replace damaged cells and proliferate sufficient osteogenic cells for preparing to form new bone tissue [21,28]. Our findings reveal that all VM-SF/CaP/MC formulations enhance MC3T3-E1 cell migration and proliferation in a dose-dependent manner within 48 h. These potential effects may be attributed to SF and CaP, which play an important role in regulating cell behavior because they provide a biocompatible environment to facilitate cell adhesion, migration, and proliferation [29]. In particular, the VM-HSF/HCaP/MC formulation showed the highest migration and proliferation rate, indicating the synergistic effect of SF and CaP. Several studies have demonstrated that SF plays a key role in the wound-healing process of bone cells [30,31]. Cheng G et al. fabricated SF/PCL nanofibrous membranes and demonstrated that SF promotes the proliferation and attachment of MC3T3-E1 cells on the membranes [32]. Additionally, Cheng, Y., et al. reported that amino acid sequences (Gly–Ser–Gly–Ala–Gly–Ala)_n_ in SF influence preosteoblast cell adhesion, migration, and proliferation [33]. Previous studies have proved that SF binds to the cell surface receptors and stimulate cell migration and proliferation via the activation of mitogen-activated protein kinase (MAPK) signaling pathway including c-Jun N-terminal kinases 1/2 (JNK1/2) and extracellular signal-regulated kinases 1/2 (ERK 1/2) [34,35,36]. In this study, CaP also showed the enhanced cell migration and proliferation of MC3T3-E1 cells. This result might be attributed to the osteoconductivity of CaP that supports cell growth [37]. All VM-SF/CaP/MC formulations demonstrated a significant dose-dependent improvement in both cell migration and proliferation. As a result, the 3% *w*/*v* concentration was determined to be the most suitable for osteogenic differentiation testing.

To assess the in vitro osteogenic differentiation, osteogenic markers, including ALP activity, collagen deposition, and mineralization, were evaluated in this study. To mimic the hydrogel post-implantation behavior at the target site and release the components, the release medium from 3% *w*/*v* VM-SF/CaP/MC was treated with MC3T3-E1 cells.

Following cell recruitment through proliferation, the MC3T3-E1 cells undergo differentiation and mineralization to form the extracellular bone matrix. During osteogenic differentiation, preosteoblasts differentiate into mature osteoblasts, evidenced by the production and the secretion of alkaline phosphatase (ALP) and type I collagen in the early stage of bone formation [38]. In addition, the process of matrix mineralization is considered the ultimate “golden” endpoint in measuring bone formation, as it signifies the late stage of osteogenic differentiation through the deposition of calcium [39].

As expected, all VM-SF/CaP/MC formulations could promote the production of ALP, collagen matrix, and calcification in MC3T3-E1 cells, indicating the progression of osteogenic differentiation. This finding suggests that SF and CaP play a crucial role in osteogenesis. Interestingly, the formulations with high SF content (VM-HSF/HCaP/MC and VM-HSF/LCaP/MC) presented higher osteogenic differentiation potential than the formulation with low SF content (VM-LSF/HCaP/MC), suggesting that SF has a significant role in osteogenic differentiation. Furthermore, SF promotes osteoblast differentiation by suppressing the Notch signaling pathway through the inhibition of Notch-specific receptor activation and downstream Notch-activated genes, which stimulates ALP activity, Col I production, and mineralization [40]. Additionally, it has been reported that SF can regulate the direction and speed of cell differentiation by interacting with intracellular signaling pathways, such as transforming growth factor beta (TGF-β) and bone morphogenetic protein (BMP) [34]. Moreover, Ca^2+^ provides an optimal environment for MC3T3-E1 cell growth, potentially affecting proliferation and promoting osteogenic differentiation. Ca^2+^ is known to regulate various cellular processes, including osteogenesis [32]. The gradual rise in extracellular calcium levels likely elevates intracellular calcium concentration in osteoblasts, influencing their functions and stimulating proliferation [41]. Furthermore, the Ca^2+^ may enter cells through calcium channels, activating the calcium/calmodulin (CaM)-mediated calcium/calmodulin-dependent protein kinase (CaMK) pathway and calcium-sensing receptors (CaSRs) along with L-type calcium channels, resulting in activation that ultimately enhances ALP activity and the production of osteopontin (OPN) and bone sialoprotein (BSP) in MC3T3-E1 cells, promoting cellular differentiation and mineralization, as reported previously [42,43].

Based on our current and previous studies, incorporating SF and CaP into hydrogel formulations has been shown to enhance osteogenic differentiation activity. Therefore, it can be concluded that all VM-SF/CaP/MC hydrogel formulations could serve as a dual-function local drug delivery system for osteomyelitis, supporting bone formation and infection treatment. Due to the potential for prolonged VM release and the eradication of bacteria for a month, it could enhance bone regeneration from the infection lesion. Furthermore, the high SF and CaP content in the hydrogel show significant potential for bone formation, as demonstrated by VM-HSF/HCaP/MC. VM-HSF/HCaP/MC provides excellent hydrogel properties with notable antibacterial and osteogenic effects, making them a promising solution for local osteomyelitis treatment and possibly opening new avenues for healing.

## 4. Conclusions

Novel VM-SF/CaP/MC thermosensitive hydrogels were successfully developed, and they exhibited excellent properties for local antibiotic administration in treating osteomyelitis. The present study further demonstrated the potential of VM-SF/CaP/MC for enhancing osteogenic differentiation in MC3T3-E1 preosteoblast cells. Based on our conducted studies, all VM-SF/CaP/MC formulations promoted both cell migration and proliferation in a dose- and time-dependent manner within 48 h. Furthermore, in vitro osteogenic differentiation studies revealed that all VM-SF/CaP/MC formulations could promote osteogenic differentiation in MC3T3-E1 cells as influenced by SF and CaP content in the formulations. Remarkably, the cells cultured in VM-HSF/HCaP/MC displayed the highest ALP production, collagen deposition, and mineralization, indicating the potential candidate for bone regeneration. Therefore, this novel VM-HSF/HCaP/MC hydrogel not only prolongs antibiotic activity but also facilitates bone repair, which offers a great potential application for osteomyelitis treatment.

## 5. Materials and Methods

### 5.1. Materials

Degummed silk yarns of Bombyx mori were supplied by Badin Thai Silk-Khorat (Nakhon Ratchasima, Thailand). Methylcellulose (MC) with a viscosity of 4000 cP, β-Glycerophosphate disodium salt hydrate (for cell culture), dexamethasone, and L-ascorbic acid were purchased from Sigma-Aldrich (St. Louis, MO, USA). Vancomycin HCl (VM) was purchased from Bio Basic Inc. (Markham, ON, Canada). Minimum essential medium alpha modification with L-glutamine, ribo- and deoxyribonucleosides (MEM-alpha), and fetal bovine serum (FBS) were purchased from Cytiva (Logan, UT, USA). Also, 0.25% Trypsin–EDTA and antibiotic–antimycotic (AB/AM) solution containing penicillin (10,000 units/mL), streptomycin (10 mg/mL), and amphotericin B (25 μg/mL), were purchased from Gibco (New York, NY, USA).

### 5.2. Extraction of Regenerated Silk Fibroin

Regenerated silk fibroin (SF) was prepared following the modified protocol from Phewchan et al. [12]. Briefly, 5 g of short silk yarns were mixed in a solution with a CaCl_2_: H_2_O: Ca(NO_3_)2: EtOH weight ratio of 30:45:5:20 and subjected to heating at 80 °C for 4 h. Subsequently, the resulting solution was dialyzed for 4 days using a 10,000 MWCO SnakeSkinTM dialysis tubing (Thermo scientific, Rockford, IL, USA) against distilled water, with daily water replacement for salt removal. Following dialysis, the SF solution was centrifuged at 10,000 rpm, at 4 °C, for 30 min to eliminate silk aggregates, and then frozen at −80 °C and subjected to lyophilization using a freeze dryer (Heto PowerDry LL3000, Thermo Fisher, Waltham, MA, USA) at 1 × 10^4^ Torr and −55 °C to obtain the regenerated SF. This regenerated SF was stored in sealed plastic bags at −20 °C until required.

### 5.3. Preparation of VM-SF/CaP/MC Hydrogels

Three different VM-SF/CaP/MC formulations, VM-HSF/LCaP/MC, VM-LSF/HCaP/MC, and VM-HSF/HCaP/MC, were prepared according to an existing method [10]. Briefly, 10 mg of VM was dissolved in 5 mL of SF solution at pH 4.8. Then, CaCl_2_ powder was added and mixed well. After that, Na_2_HPO_4_ aqueous solution was added dropwise under continuous stirring to produce low CaP (LCaP) and high CaP (HCaP) content with final CaCl_2_:Na_2_HPO_4_ molar ratios of 0.3:0.2 and 0.5:0.3, respectively. Finally, MC was directly added to the mixture solution under continuous stirring for 30 min, as illustrated in Scheme 1a. The final product contained low SF (LSF) and high SF (HSF) content of 1.5 and 2% *w*/*v*, while MC was maintained at 2% *w*/*v*. Finally, all VM-SF/CaP/MC solutions were kept at 4 °C before further investigation. The compositions of different VM-SF/CaP/MC formulations are presented in Table 1.

### 5.4. Cell Culture and Specimen Preparation

The cytocompatibility, cell proliferation, cell migration, and osteogenic differentiation potential of VM-SF/CaP/MC were evaluated using Murine calvarial preosteoblast cell line subclone 14 (MC3T3-E1; CRL-2594, ATCC, Manassas, VA, USA). MC3T3-E1 cells were cultured in complete growth medium of MEM-alpha medium supplemented with 10% FBS and 1% AB/AM at 37 °C in a 5% CO_2_ humidified atmosphere. The culture medium was refreshed every 2 days. At 80% confluence, the cells were then trypsinized and seeded into culture well plates for further experimentation.

All VM-SF/CaP/MC hydrogel formulations were prepared under aseptic conditions following a sample extraction protocol according to international standard ISO10993-5:2009 guidelines, which have established procedures for testing the in vitro cytotoxicity of medical devices [18]. Briefly, the various amounts of all VM-SF/CaP/MC solutions at 0.05, 0.10, 0.15, 0.20, and 0.25 g were solidified to form a hydrogel at 37 °C for 1 h. Afterward, they were incubated in 5 mL serum-free medium at 37 °C for 24 h with shaking at 50 rpm, representing VM-SF/CaP/MC concentrations of 1–5% *w*/*v* in medium. Subsequently, samples were centrifuged at 6000 rpm for 15 min, and the supernatants of all VM-SF/CaP/MC formulations were collected as the extract solutions of VM-SF/CaP/MC for cell viability, cell migration, and cell proliferation experiments.

### 5.5. Cell Viability Assay

To assess the optimal concentration of each VM-SF/CaP/MC for in vitro osteogenic differentiation studies, a quantitative cell viability assay was conducted using a cell counting kit-8 (CCK-8, DOJINDO, Kumamoto, Japan). The CCK-8 is a colorimetric assay involving the reduction of tetrazolium salt WST-8 by dehydrogenases in viable cells, resulting in the formation of water-soluble orange formazan dye.

MC3T3-E1 cells were seeded in 96-well plates at 1 × 10^4^ cells/well and pre-cultured for 24 h. The cells were treated with 100 μL of extract solution from the VM-SF/CaP/MC gels prepared with concentrations of 1–5% *w*/*v* in medium, incubated at 37 °C for 24 h. After 24 h of incubation, the treated cells were washed twice with PBS. A 10% *v*/*v* of CCK-8 solution was subsequently added to each well and incubated in the dark at 37 °C for 2 h. Absorbance at 450 nm was measured using a microplate reader (Synergy H1 Hybrid Reader, Agilent BioTek, Santa Clara, CA, USA). The control group consisted of cells cultured in serum-free medium (cell viability 100%).

### 5.6. Cell Migration Assay

Cell migration was explored using an in vitro scratch wound-healing assay. Briefly, MC3T3-E1 cells were seeded at 5 × 10^4^ cells/well in 24-well culture plates and pre-cultured for 24 h until reaching 80% confluence. Then, the damaged wound area was created by interrupting the cell monolayer using a 200 μL pipette tip. The debris cells were removed by washing with sterile PBS. Subsequently, the cells were treated with 500 μL of extract solution from the VM-SF/CaP/MC gels at 1, 2, and 3% *w*/*v*, which were prepared in complete growth medium for 24 and 48 h. The control cells were cultured in complete growth medium without the sample. After the scratch at 0, 24, and 48 h, the cells were fixed in 4% *v*/*v* paraformaldehyde (PFA) for 30 min and stained with 0.1% *w*/*v* crystal violet (CV) (Riedel-de Haën, Munich, Germany). At least three images of the scratched area were photographed under a bright-field light microscope (ZEISS Axio Observer.Z1, White Plains, NY, USA). The number of migrated cells was counted, and the percentage of scratch closure was quantified using Image J (version 1.54k) software. The same scratched area was selected for investigation at each time point of the study, and the experiment was performed in triplicate.

### 5.7. Cell Proliferation Assay

Cell proliferation was examined using a CCK-8 assay and CV staining. MC3T3-E1 cells were seeded in 96-well plates at 5 × 10^3^ cells/well and pre-cultured for 24 h. The cells were then treated with 100 μL of extract solution from the VM-SF/CaP/MC gels at 1, 2, and 3% *w*/*v*, which were prepared in complete growth medium for 24 and 48 h. The control cells were cultured in a complete growth medium without the sample. For the quantitative analysis, CCK-8 solution was added to each well, incubated in the dark at 37 °C for 2 h, and the absorbance was measured at 450 nm using a microplate reader. For a qualitative assessment, the treated cells were fixed with 4% PFA, stained with 0.1% *w*/*v* CV, and observed under a bright-field light microscope to investigate the cell density and morphology.

### 5.8. Evaluation of Osteogenic Differentiation

The effect of VM-SF/CaP/MC on the osteogenic differentiation of MC3T3-E1 cells was evaluated by exposing the cells to the release medium from VM-SF/CaP/MC for 35 days. This setup simulates the practical scenario in which hydrogel implants deliver antibacterial agents at infection sites and gradually degrade over time. A 3% *w*/*v* concentration of each hydrogel formulations was transformed into a gel formation at 37 °C for 1 h, followed by incubation in 5 mL of serum-free medium at 37 °C for 35 days. The release medium was collected every other day and replaced with 5 mL of fresh serum-free medium. The collected medium was then combined with osteogenic medium for testing, supplemented with 10% FBS, 1% AB/AM, 50 μM L-ascorbic acid, 10 mM β-glycerophosphate, and 100 nM dexamethasone, referring to the sample medium. A medium without the released components of VM-SF/CaP/MC served as the control.

MC3T3-E1 cells were seeded in 24-well plates at a density of 5 × 10^4^ cells/well and cultured in complete growth medium for 24 h. The complete growth medium was then replaced with 500 µL of the sample medium. The cells were incubated at 37 °C in a 5% CO_2_ atmosphere for 35 days, and the sample medium was refreshed every three days. At determined times, the effect of the sample medium on osteogenic differentiation was evaluated by measuring alkaline phosphatase (ALP) activity, staining with Sirius Red (SR) for collagen deposition and Alizarin Red S (ARS) for mineralization.

#### 5.8.1. Alkaline Phosphatase Activity

Alkaline phosphatase (ALP) activity was quantified at 7 and 14 days of the treatment using an Alkaline Phosphatase Colorimetric Assay Kit (ab83369; Abcam, Boston, MA, USA), following the manufacturer’s instructions. Briefly, MC3T3-E1 cells were lysed in 50 μL of assay buffer and homogenized on ice. The lysate cells were centrifuged at 4 °C at 12,000 rpm, and the supernatant of each sample was collected. Then, 20 μL of supernatant was mixed with 50 μL of 5 mM p-nitrophenyl phosphate (p-NPP) substrate in 96-well plates and incubated for 60 min in the dark at room temperature. The reaction was terminated through the addition of 20 μL of stop solution, and the absorbance of the formed p-nitrophenol (p-NP) was measured at 405 nm using a microplate reader. In addition, the total intracellular protein content was determined using a BCA Protein Assay Reagent Kit (Thermo Fisher Scientific, Rockford, IL, USA). The ALP activity was subsequently normalized to the corresponding total protein content and expressed as U/mg protein. Each experiment was performed in triplicate.

#### 5.8.2. Collagen Deposition

Collagen deposition in MC3T3-E1 cells was evaluated by Sirius Red (SR) staining modified from originally described by Tullberg-Reinert [44]. At each time point, the cells were washed twice with PBS and fixed in 4% *v*/*v* PFA for 30 min. MC3T3-E1 cells were then stained with 0.1% *w*/*v* Sirius Red (SR) (Sigma-Aldrich (St. Louis, MO, USA)) in a saturated picric acid solution for 1 h. Subsequently, the stained cells were washed with 0.1 M acetic acid to remove non-specifically bound dye, air-dried at room temperature, and visualized collagen deposition under an inverted phase-contrast microscope (ZEISS Axio Observer Z1, White Plains, NY, USA).

For quantitative analysis, the stained cells in each well were eluted by the destain solution (0.2 M NaOH–methanol, 1:1), and then the solutions were centrifuged at 12,000 rpm for 30 min. OD was measured at 540 nm using a microplate reader. The collagen content was calculated using the standard curve of type I collagen in 0.05 M acetic acid with a concentration range of 5–80 μg/mL.

#### 5.8.3. Extracellular Matrix Mineralization

For the matrix mineralization assessment, the cells were washed twice with PBS and fixed with 70% *v*/*v* ethanol for 1 h. Then, the treated cells were stained with 40 mM Alizarin Red S (ARS) (Sigma-Aldrich (St. Louis, MO, USA)) at pH 4.2 for 20 min at room temperature, followed by extensively rinsing with ultrapure water until the red coloration disappeared, and dried at room temperature. Mineralized nodules were visualized using an inverted phase-contrast microscope, and the total calcium content was extracted by adding 0.5 mL of 10% *w*/*v* cetylpyridinium chloride in 10 mM Na_2_HPO_4_ (pH 7) for 2 h at 37 °C for quantitative analysis. Subsequently, 200 µL of all samples was transferred to a 96-well plate, and the OD was measured at 620 nm using a microplate reader. The calcium content was calculated using a calibration curve of standard ARS in the concentration range of 0.03–2.00 mM.

### 5.9. Statistical Analysis

Data analysis was conducted using GraphPad Prism^®^ 10.0.3 software (GraphPad Software, La Jolla, CA, USA). Statistical significance was determined using one-way ANOVA with Tukey’s post hoc test, with a *p*-value of less than 0.05 (*) considered statistically significant. Data are presented as mean ± SD (standard deviation), and the number of replicates is indicated below each figure. The microscopic analyses were observed using a triple-investigation area.

## Figures and Tables

**Table 1:** The compositions of VM-SF/CaP/MC hydrogels.

Compositions	VM-SF/CaP/MC		
	VM-HSF/LCaP/MC	VM-LSF/HCaP/MC	VM-HSF/HCaP/MC
SF content (% *w*/*v*)	2.0	1.5	2.0
CaCl_2_:Na_2_HPO_4_ molar ratio	0.3:0.2	0.5:0.3	0.5:0.3
MC content (% *w*/*v*)	2.0	2.0	2.0

## Data Availability

The raw data supporting the conclusions of this article will be made available by the authors on request.
